# Phase II open‐label multicenter study to assess the antitumor activity of afatinib in lung cancer patients with activating epidermal growth factor receptor mutation from circulating tumor DNA: Liquid‐Lung‐A

**DOI:** 10.1111/1759-7714.13763

**Published:** 2020-12-03

**Authors:** Cheol‐Kyu Park, Sung‐Yong Lee, Jae Cheol Lee, Chang‐Min Choi, Shin Yup Lee, Tae‐Won Jang, In‐Jae Oh, Young‐Chul Kim

**Affiliations:** ^1^ Department of Internal Medicine Chonnam National University Medical School and CNU Hwasun Hospital Hwasun Jeonnam Korea; ^2^ Department of Internal Medicine Korea University Guro Hospital Seoul Korea; ^3^ Department of Oncology, Pulmonary and Critical Care Medicine College of Medicine, University of Ulsan, Asan Medical Center Seoul Korea; ^4^ Department of Internal Medicine, School of Medicine Kyungpook National University Daegu Korea; ^5^ Department of Internal Medicine, School of Medicine Kosin University Gospel Hospital Pusan Korea

**Keywords:** Afatinib, circulating tumor DNA, *EGFR* mutation, non‐small cell lung carcinoma

## Abstract

**Background:**

Mutation analysis of circulating tumor DNA (ctDNA) is used for diagnosing lung cancer. This trial aimed to assess the efficacy of afatinib in treatment‐naïve patients with lung cancer harboring epidermal growth factor receptor mutations (EGFRm, exon 19 deletions or exon 21 point mutations) detected based on ctDNA.

**Methods:**

The primary objective was the objective response rate (ORR) in the response evaluable (RE) population. EGFRm analysis of ctDNA was performed using PANA Mutype. Of the 331 patients screened, ctDNA was positive in 21% (68/331) in the detection of activating EGFRm. Among 81 subjects with tumor EGFRm, 48 showed matched EGFRm in their ctDNA (59% sensitivity).

**Results:**

Therapy with afatinib 40 mg was initiated in 21 (female, 17; adenocarcinoma, 20) patients (intention‐to‐treat, ITT); dose modifications were made in 15 (71%). The ORR was 74% in the RE population (14/19); 11 patients showed EGFRm only in ctDNA (Group A), whereas 10 exhibited the same EGFRm in their ctDNA and tumor DNA (Group B). There was no significant difference in ORR between Groups A and B (80% and 67% RE, respectively). Median progression‐free survival (PFS) was 12.0 months, and no significant difference was observed according to the final afatinib dose, type of EGFRm, and Group A versus B. After progression, T790M mutation was found in 40% (6/15) of patients, and osimertinib was used as a second‐line treatment.

**Conclusions:**

Afatinib showed similar ORR and PFS in patients with lung cancer harboring EGFRm in their ctDNA regardless of tumor EGFRm results.

**Key points:**

**Significant findings of the study:**

Afatinib showed favorable ORR and PFS regardless of the tumor *EGFR* mutation status results, similar to the findings of previous trials assessing afatinib as first‐line treatment of *EGFR*‐mutated non‐small cell lung cancer based on tumor genotyping.

**What this study adds:**

Our findings emphasize that the survival benefit of afatinib treatment can be achieved not only by appropriate dose reduction with frequent and detailed monitoring of toxicities, but also by using noninvasive (ctDNA) assays in a real‐world setting.

## Graphical abstract

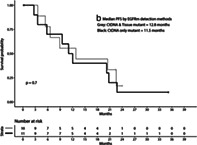

Afatinib showed favorable ORR and PFS regardless of the tumor *EGFR* mutation status results, similar to the findings of previous trials assessing afatinib as first‐line treatment of *EGFR*‐mutated NSCLC based on tumor genotyping. The survival benefit of afatinib treatment can be achieved not only by appropriate dose reduction with frequent and detailed monitoring of toxicities, but also by using noninvasive (ctDNA) assays in a real‐world setting

## Introduction

Lung cancer is the leading cause of cancer‐related death in Korea.^1^ The most common histological type of lung cancer is adenocarcinoma (48%), and 37% of these patients have activating epidermal growth factor receptor mutations (EGFRm).^2^ Almost half (45%) of the patients with non‐small cell lung cancer (NSCLC) are diagnosed at stage IV^2^ and require palliative medical treatments. Among the first‐line options for stage IV NSCLC with activating EGFRm, afatinib has shown better treatment efficacy than platinum doublets^3^, ^4^ and gefitinib.^5^ Dacomitinib,^6^ another second‐generation EGFR‐tyrosine kinase inhibitor (EGFR‐TKI), showed longer overall survival (OS) than gefitinib. Osimertinib,^7^ a third‐generation EGFR‐TKI, was proven superior to first‐generation TKIs; however, the best treatment sequence in Asian patients is still controversial.^7^

Knowledge of the tumor driver mutation status is critical when selecting the best treatment regimen for stage IV NSCLC. Obtaining adequate tumor tissue or cytological samples is not always possible. Previous studies have shown that circulating tumor DNA (ctDNA) can be used as a suitable substitute for tissue biopsy sample for mutation analysis. The sensitivity of detecting activating EGFRm using ctDNA has been reported in the range of 66% to 75%,^8^, ^9^ or even 100%,^10^ depending on the testing platform used.

Studies have reported similar efficacy between EGFR‐TKIs in patients with activating EGFRm in their ctDNA.^11^, ^12^ In a Spanish trial of 1033 NSCLC patients without biopsy samples or with insufficient tumor tissue, activating EGFRm were found in the ctDNA of 113 patients (11%) using real‐time polymerase chain reaction (RT‐PCR). An objective response rate (ORR) of 72% and median progression‐free survival (PFS) of 11 months were observed in 18 patients treated with first‐line EGFR‐TKIs based on their ctDNA results.^11^ This trend was reproduced in a Chinese trial, which used droplet digital PCR (ddPCR). Among the 426 patients screened, 188 with EGFRm in their ctDNA received gefitinib, and the ORR was 72.1%, whereas the median PFS was 9.5 months.^12^ Thus, first‐line EGFR‐TKI treatment based on ctDNA EGFRm results has already been approved by the European Medicines Agency, the US Food and Drug Administration (FDA), and many other countries. This trial was designed to confirm the treatment efficacy of afatinib in patients with NSCLC harboring EGFRm detected in ctDNA.

## Methods

### Study design and subjects

This trial, called Liquid‐Lung‐A, was a phase II, open‐label, prospective multicenter study conducted at five centers in South Korea. Patients with stage IIIB to IV NSCLC, aged >18 years, and with Eastern Corporative Oncology Group (ECOG) performance status 0 to 2 were included. Activating EGFRm (exon 19 deletion, L858R, L861Q, G719X) was detected in ctDNA using PNA‐based RT‐ PCR as described below. Patients were expected to have at least one measurable lesion that was not previously irradiated, defined using the Response Evaluation Criteria in Solid Tumors (RECIST) version 1.1.^13^ The exclusion criteria are provided in detail in the Supporting Information (SuppInfo Data_TCA.doc). Patients with central nervous system (CNS) metastases were enrolled if their disease was asymptomatic or stable after local therapy, including surgery or radiotherapy, before the first dose of afatinib.

This trial was performed in accordance with the Declaration of Helsinki and Good Clinical Practice guidelines and was approved by the institutional review board of the relevant institution (CNUHH‐2016‐025) and the Korean Ministry of Food and Drug Safety (30837). All patients were required to provide written informed consent before participating in this study. This trial is registered at the clinical trials registry (ClinicalTrials.gov Identifier: NCT02629523).

### Plasma and tumor EGFRm tests

All patients were required to provide a blood sample at screening to test for activating EGFRm in the plasma. To this end, 10 mL blood samples were withdrawn, centrifuged at 4°C to obtain the plasma (for 10 minutes at 2000 × *g* to remove cells, and then for 10 minutes at 3000 × *g* using the supernatant to deplete platelets), and then stored at −20°C until delivery to the Central Laboratory (Panagene Inc., Daejeon, South Korea). EGFRm tests for ctDNA were performed at the Central Laboratory using a PANA Mutyper R EGFR assay (Panagene Inc.). In‐house EGFRm testing for tumor DNA was performed by each hospital using a PNA clamp EGFRm or PANA Mutyper EGFR kit (Panagene Inc.).

### Trial procedure, assessment, and treatment

Eligible patients received 40 mg afatinib once a day until disease progression, as defined using RECIST v1.1,^13^ unacceptable toxicity, or any other valid reason for ceasing treatment occurred. Responses were evaluated every eight weeks for the first three assessments (24 weeks), followed by every 12 weeks for subsequent assessments. Regular brain imaging was performed in patients with known brain metastases. For the rest of the patients, brain imaging was only performed when there were symptoms or signs of suspected CNS metastases. Patients could continue afatinib after RECIST v1.1‐defined progression if they maintained clinical benefits, as assessed by an investigator.

The primary objective of the study was to determine the ORR (assessed using RECIST v1.1) to afatinib in EGFR‐TKI‐naïve NSCLC patients with activating *EGFR* mutations detected in ctDNA. The secondary objective was the determination of the PFS, OS, and safety profile. PFS was defined as the time (months) from the first dose of afatinib until objective disease progression or death, regardless of whether the patient was withdrawn from therapy or received another anticancer therapy before progression. OS was defined as the time (months) from the first dose of afatinib to the death of the patient.

Adverse events (AEs) were measured from the beginning of drug administration, throughout the treatment period, until 28 days after the last dose of afatinib. AEs were graded according to the National Cancer Institute Common Terminology Criteria for Adverse Events (CTCAE), version 4.0. If a patient experienced diarrhea of CTCAE grade 2 (>2 days) or higher that was considered to be associated with afatinib, dosing could be interrupted for up to two weeks. If the diarrhea resolved or reverted to CTCAE grade ≤ 1 within two weeks of onset, afatinib could be restarted at a lower dose (30 or 20 mg, daily). For other toxicities, afatinib could be restarted at the same or lower dose if the toxicities resolved or reverted to CTCAE grade ≤ 1, excluding cases with any grade of pulmonary toxicity. Once a dose was reduced, it was not increased in future cycles.

### Statistical analysis

**Figure 1:**
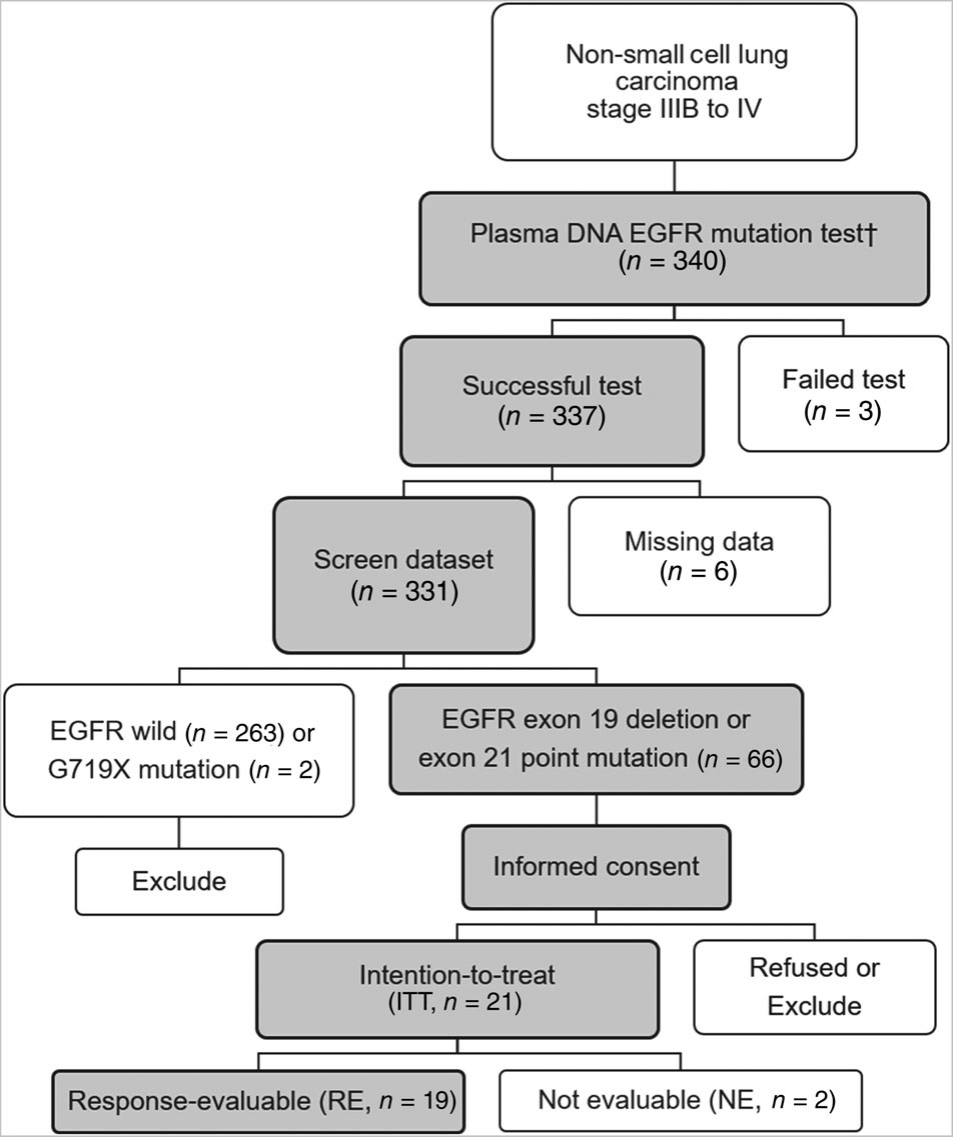
CONSORT diagram of study participants. †Tumor histology of the screened patients were as follows: adenocarcinoma, *n* = 298; squamous cell carcinoma, *n* = 21; others, *n* = 9; without tissue diagnosis, *n* = 12

The response rate to afatinib treatment was assumed to be at least 50% compared to the 25% in EGFRm‐negative patients or those with unknown EGFRm status. To prove a >50% response rate to afatinib using this study design with a statistical power of 80%, 21 patients were needed. The baseline characteristics and safety data were analyzed in an intention‐to‐treat (ITT) population, which was defined as the patients who had received at least one dose of the treatment and for whom AEs were monitored (*n* = 19, Fig 1). According to the study protocol, the ORR was assessed in the response evaluable (RE) population, defined as patients who had received at least one treatment dose and for whom response evaluations were available. The PFS and OS were analyzed in the ITT population.

Intergroup comparisons were performed using the Mann–Whitney U test for continuous variables and Pearson's χ^2^ or Fisher's exact tests for categorical variables. Survival times were estimated for each group using the Kaplan–Meier method. Statistical analyses were performed using R statistics,^14^ and *P‐*values <0.05 were considered statistically significant.

## Results

### Sensitivity of tests for detecting activating EGFRm in ctDNA

**Figure 2:**
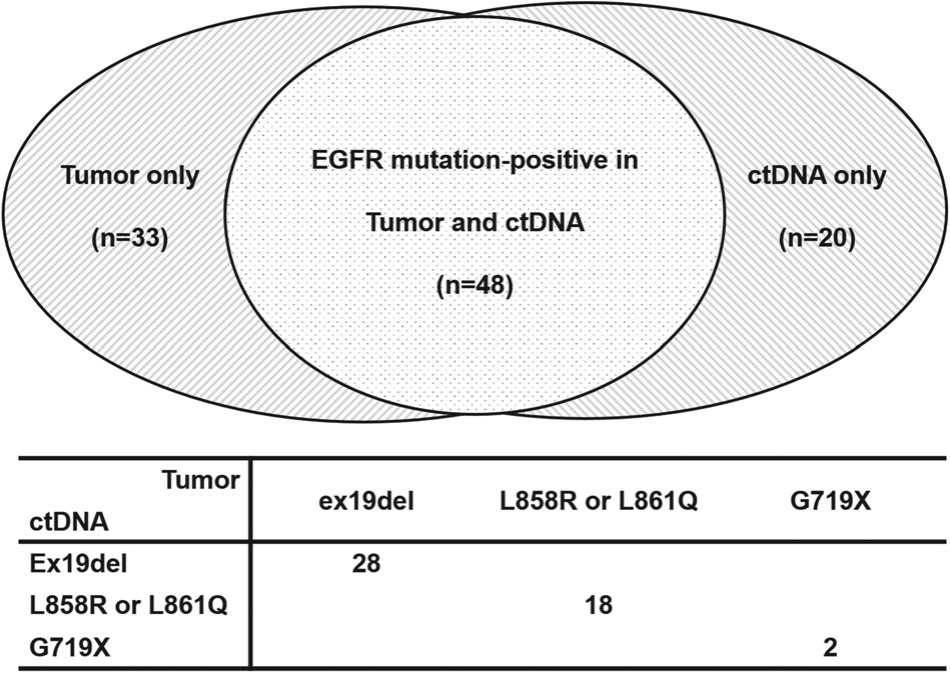
Results of tumor and circulating tumor DNA (ctDNA) epidermal growth factor receptor mutation (EGFRm) tests and concordance of EGFRm between tumor DNA and ctDNA. Ex19del, exon 19 deletion.

Among the 340 patients screened (adenocarcinoma, 298; squamous cell carcinoma, 21; others, nine; without tissue diagnosis, 12), 331 patients were successfully tested for ctDNA EGFRm without missing data from December 2016 to March 2018 (Fig 1). Tumor and ctDNA genotyping showed 24% (81/331) and 21% (68/331) positive detection of activating EGFRm, respectively (exon 19 deletions, exon 21 point mutations, and G719X). Among the 81 tumor DNA EGFRm‐positive patients, 48 showed EGFRm in their ctDNA (59% sensitivity). The EGFRm types were completely matched between the tumor DNA and ctDNA in 48 patients (Fig 2).

### Patient characteristics

Among the 68 patients with activating EGFRm in their ctDNA, 21 were enrolled in this trial, and their clinical characteristics are summarized in Table 1. Afatinib was initiated at a daily dose of 40 mg in 21 ITT patients with a mean age of 68.5 years. Most patients (20/21) had adenocarcinoma, 81% were women, and 33% had brain metastases upon enrollment in this trial. Dose modifications were made in 15 (71%) patients.

**Table 1:** Patient characteristics

	Intention‐to‐treat (*n* = 21)	Group A: Only in ctDNA (*n* = 11)	Group B: Both ctDNA and tumor DNA (*n* = 10)
Age, years	68.5 ± 8.7	70.5 ± 7.8	66.3 ± 9.5
Sex, female/male	17/4	9/2	8/2
Height, cm	154.3 ± 7.6	152.5 ± 8.4	156.2 ± 6.5
Weight, kg	56.0 ± 8.2	55.8 ± 7.0	56.3 ± 9.9
Stage IV	21	11	10
PS (ECOG)†, 0/1/2	8/11/2	2/7/2	6/4/0
Adenocarcinoma/NSCLC, NOS	20/1	10/1	10/0
Brain metastases before treatment, Yes/No	7/14	4/7	3/7
Final dose, 20/30/40 mg	6/9/6	4/3/4	2/6/2
Dose reduction, Yes/No	15/6	7/4	8/2
Type of *EGFR* mutation, ex19del/ex21pm	12/9	7/4	5/5
Response, PR/SD/PD/NE	14/5/0/2	8/2/0/1	6/3/0/1
CNS progression, Yes/No^‡^	3/15	1/9	2/6
T790M after PD, Yes/No/ND^‡^	6/9/3	3/4/3	3/5/0
Subsequent treatment, osimertinib/others^§^/ BSC/afatinib ongoing	6/8/4/3	3/4/3/1	3/4/1/2

Values are presented as the mean ± standard deviation or number.

^†^ Performance status score using Eastern Corporative Oncology Group.

^‡^ Counted among subjects with disease progression after afatinib treatment (n = 18), and tumor rebiopsy and circulating tumor DNA were used for EGFR T790M analyses.

^§^ Chemotherapy, immunotherapy, gefitinib or combined.

BSC, best supportive care; CNS, central nervous system; ctDNA, circulating tumor DNA; EGFR, epithelial growth factor receptor; ex19del, exon 19 deletion; ex21pm, exon 21 point mutation; ND, not done; NE, not evaluable; NOS, not otherwise specified; NSCLC, non‐small cell lung carcinoma; PD, progressive disease; PR, partial response; SD, stable disease.

Eleven patients showed EGFRm only in the ctDNA (tumor DNA EGFR wild‐type or unknown, Group A) and 10 exhibited the same EGFRm in both their ctDNA and tumor DNA (Group B, Table 1). Groups A and B showed no significant differences in age, sex, height, weight, performance status, histology, dose reduction rates of afatinib, and type of EGFRm.

### Response

**Figure 3:**
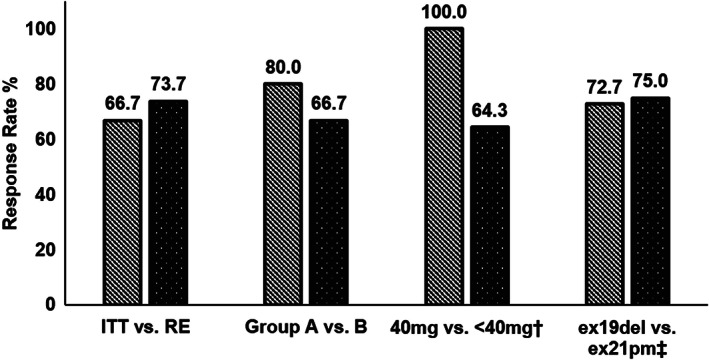
Comparison of the response rate according to the subgroups. All results were calculated in the RE population. †Final dose of afatinib, ‡Type of *EGFR* mutation (EGFRm). EGFR, epidermal growth factor receptor; ITT, intention‐to‐treat; RE, response evaluable; ex19del, exon 19 deletion; ex21pm, exon 21 point mutation.

Response evaluation was feasible in 19 patients and the remaining two stopped treatment before response evaluation. One patient discontinued treatment on day 8 because of drug‐ induced interstitial lung disease and showed EGFR exon 21 point mutation in ctDNA without available tumor tissue (Group A). Another patient, who withdrew consent on day 37 because of grade 3 mucositis, had an EGFR exon 19 deletion in both ctDNA and tumor DNA (Group B). A partial response was observed in 14 patients, and the ORR was 67% and 74% in the ITT and RE populations, respectively. There was no significant difference (*P* = 0.35) in the ORR between Groups A and B (RE, 80% and 67%, respectively, Table 1). Comparisons of the response rates (Fig 3) showed no significant differences according to subgroups of the final dose (5/5 vs. 9/14, *P* = 0.26) and type of EGFRm (8/11 vs. 6/8, *P* = 0.91).

### Survival

**Figure 4:**
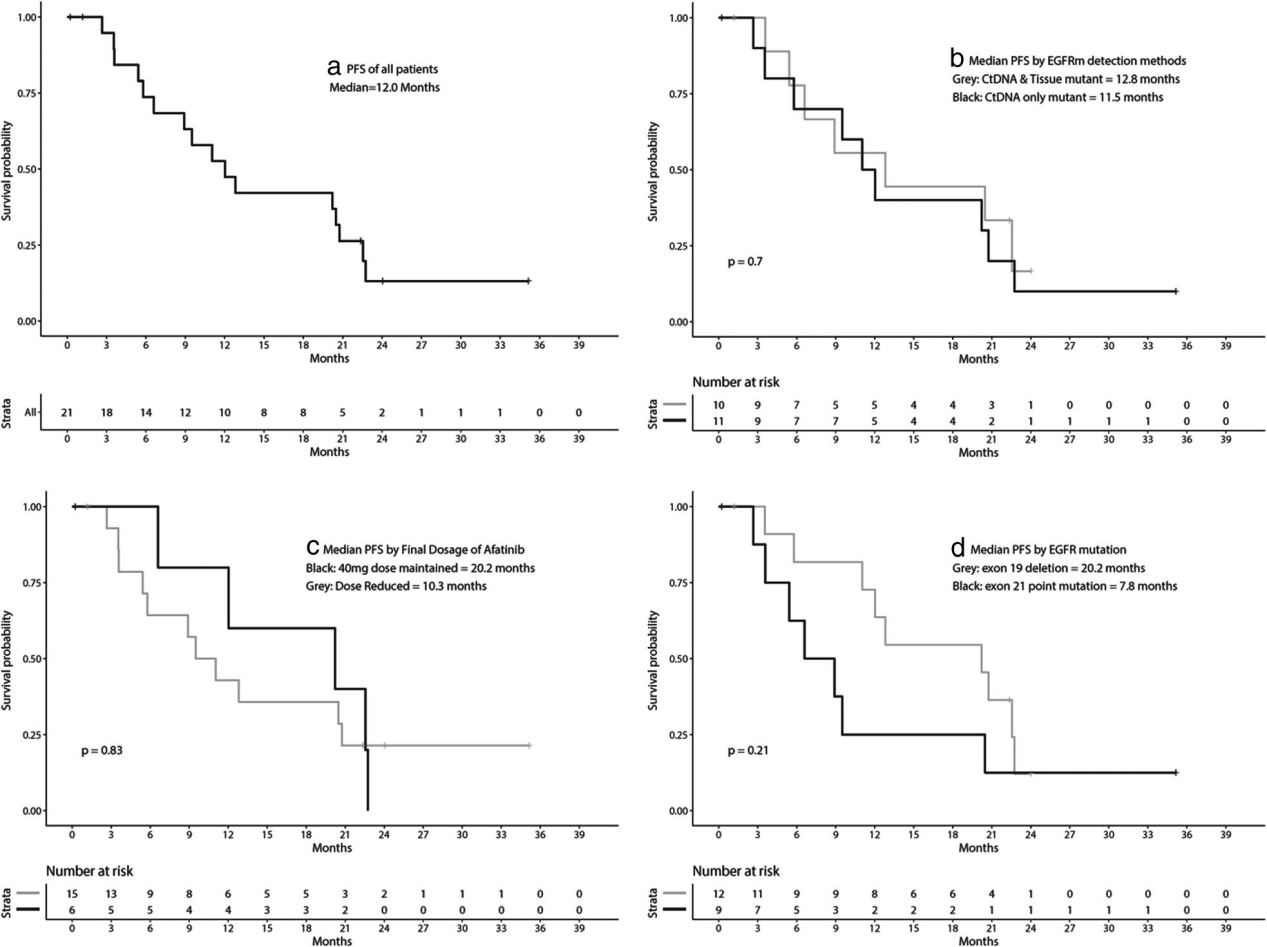
Progression‐free survival (PFS) of all patients (a) and comparisons of survival according to Group A (*EGFR* mutation only in ctDNA) versus Group B (*EGFR* mutation in ctDNA and tumor DNA) (b); final dose of afatinib (c); and type of *EGFR* mutation (d). EGFRm, epidermal growth factor receptor mutation; ctDNA, circulating tumor DNA

The median duration of follow‐up was 24.0 months, and PFS events occurred in 16 participants (76%) with a median PFS of 12.0 months (95% confidence interval [CI]: 8.9–22.8). Groups A and B showed no significant difference in the median PFS (11.5 vs. 12.8 months, log rank *P* = 0.70), which also did not significantly differ between the final dose subgroups (40 mg vs. <40 mg, 20.2 vs. 10.3 months, *P* = 0.83). The median PFS of the subgroup with an exon 19 deletion was longer (20.2 months) than that of the subgroup with an exon 21 point mutation (7.8 months, *P* = 0.21, Fig 4). At the data cutoff, the OS was not yet matured, and only 11 events occurred (52%). The median OS was 27.9 months (95% CI: 16.1–not calculated). There was no significant difference in OS between Groups A and B, the final dose subgroups, and the type of EGFRm.

### Toxicity and safety

Afatinib treatment continued in three patients until May 2020; one and two patients withdrew consent because of grade 3 mucositis and grade 3 and 5 drug‐induced interstitial lung disease, respectively. A summary of the toxicity profiles of all enrolled patients who received afatinib is shown in Table 2. Eighteen serious AEs including two cases of drug‐induced lung disease were reported. A reduction in the daily dose of afatinib was permitted independently by the investigators according to the grade of AEs. Diarrhea, skin rash, and mucositis were the main reasons for dose reductions in 15 subjects (71%).

**Table 2:** List of adverse events in 21 intention‐to‐treat (ITT) participants

	Any grade (1/2/3/5)	Grade ≥ 3
Diarrhea	8/6 /4	4 (19%)
Vomiting	0/0/1	1 (5%)
Skin rash	8/4/2	2 (10%)
Mucositis	4/3/3	2 (10%)
Paronychia	8/3/1	1 (5%)
Pneumonitis	0/0/1/1	2 (10%)
QT prolongation	0/0/1	1 (5%)
Any SAE	*n* = 18	Acute kidney injury (1), Diarrhea (2), Pleural effusion (1), Drug‐induced lung disease (2), Dyspnea (2), Back pain (1), Urinary tract infection (1), Brain metastases (1), Disease progression (7)

SAE, serious adverse events.

### Pattern of progression and subsequent treatment after osimertinib

Among the 18 patients with disease progression until data cutoff, 20% (3/15) showed disease progression in the CNS. After progression, rebiopsy or ctDNA analysis of the T790M resistance mutation or both were performed in 15 patients. The T790M mutation was found in 40% (6/15) of patients, who were then administered osimertinib as second‐line treatment. For the remaining patients, cytotoxic chemotherapy, immune checkpoint inhibitors, and first‐generation EGFR‐TKIs were used (Table 1).

## DISCUSSION

This was a prospective, multicenter study evaluating the treatment efficacy of afatinib in previously untreated patients with advanced or metastatic NSCLC harboring an EGFR activating mutation in ctDNA. In the present study, afatinib showed favorable ORR and PFS regardless of the tumor EGFRm status results, similar to the findings of previous trials assessing afatinib as first‐line treatment of EGFR‐mutated NSCLC based on tumor genotyping.^3^, ^4^, ^5^

In this trial, the detection rate of ctDNA *EGFR* activating mutations at the screening of NSCLC was higher (21%) than that reported in the previous Spanish trial (11%).^11^ However, the ctDNA test conducted in this study (Mutyper) showed a favorable but relatively lower sensitivity (59%) than that reported in previous studies,^8^, ^9^, ^10^ which was possibly due to the central delivery system not using a cell‐free DNA stabilization tube for blood collection, and the use of a less sensitive detection technique compared with ddPCR or BEAMing. Tumor EGFR genotyping in the present study showed a considerably lower prevalence rate of EGFRm (24%, 81/331) than that observed with a survey of a nationwide registry in Korea (36%).^2^

The baseline and clinical characteristics of the enrolled patients showed no significant difference between Group A (EGFRm‐positive only in ctDNA) and B (positive in both ctDNA and tumor DNA). Six of the 11 patients in Group A did not have tumor EGFRm test results because of tissue shortage. The tumor genotyping of five other patients showed a wild‐type *EGFR* gene, and these negative results could be linked to a low amount of tumor DNA, pre‐ or post‐analytic error, or spatial heterogeneity of the tumor. However, afatinib showed definite clinical efficacy in all five patients (four partial response and one stable disease). All patients in Group A could have benefited from the plasma EGFR test in this trial.

In this study, the types of EGFRm were completely concordant between the tumor DNA and ctDNA when both were positive for an *EGFR* activating mutation. A study by Oxnard *et al*.^15^ highlighted that a well‐validated assay has negligible false positives, where the clinical outcome would be the best reference standard for a noninvasive assay. Therefore, the detection of ctDNA EGFRm could reflect the accurate molecular status of the primary tumor, and the plasma EGFR test could be a useful screening tool for the diagnosis of NSCLC when tumor tissue is insufficient or tumor genotyping is not feasible. Nevertheless, because ctDNA testing has lower sensitivity than tumor genotyping, an EGFRm test using tumor tissue specimens should be conducted when an *EGFR* mutation is not detected in ctDNA.

In the present study, afatinib showed favorable efficacy in patients with NSCLC harboring EGFRm in their ctDNA regardless of the result of tumor genotyping. Groups A and B could be considered to have presented with the same EGFRm‐shedding tumor, so it is not surprising that there was no significant difference in the ORR and PFS between these groups. Comparisons of the efficacy of afatinib between previous trials (LUX‐Lung 3, 6, and 7) and our present study are summarized in Table 3. Despite unfavorable conditions such as tumor shedding (ctDNA EGFRm‐positive), relatively advanced age (mean age, 68.5 years), and the presence of brain metastases (33%), the present trial showed ORR and PFS similar to those of previous trials where patients were recruited based on tumor EGFR genotyping. In the recently published BELIEF trial,^16^ activating EGFRm was detected in ctDNA in 55 of 91 (60%) patients with tumor EGFRm, and the patients with ctDNA EGFRm showed a correlation with a shorter PFS compared to ctDNA EGFRm‐negative patients. In a biomarker analysis of the NEJ026 phase III study comparing erlotinib plus bevacizumab combination therapy with erlotinib monotherapy in *EGFR*‐mutated NSCLC,^17^ patients without ctDNA EGFRm at baseline had longer PFS compared to patients with ctDNA EGFRm. In addition, patients who showed a negative conversion of ctDNA EGFRm from baseline to six weeks after the start of treatment had longer PFS compared with patients who had ctDNA EGFRm continuously detected after six weeks. However, the present trial did not implement a comparison of treatment outcomes between ctDNA EGFRm‐positive and ‐negative patients in its protocol, and a direct comparison of the PFS in ctDNA EGFRm‐positive patients between the present trial and the BELIEF trial could be controversial.

**Table 3:** Summary of comparisons between previous trials and the present study

	LUX‐Lung 3^3^	LUX‐Lung 6^4^	LUX‐Lung 7^5^	Present trial	Spanish trial^11^	BENEFIT^12^
Type of *EGFR* mutation	All	All	e19del, e21pm	e19del, e21pm	e19del, e21pm	e19del, e21pm
EGFR‐TKI	Afatinib	Afatinib	Afatinib	Afatinib	All 1G	Gefitinib
ORR, %	56^†^ (69)^‡^	67^†^ (74)^‡^	70^†^	74^†^	72	72
Median PFS^†^, months	11.1^§^ (13.6)^¶^	11.0^§^ (11.0)^¶^	11.0^¶^	12.0^¶^	11.0	9.5
Dose reduction rate (afatinib), %	53	28	39	71	‐	‐

† Independent assessment.

^‡^ Investigator assessment.

^§^ Any type of activating mutation.

¶ Common types of activating mutation (exon 19 deletion or exon 21 point mutation).

1G, first generation; e19del, exon 19 deletion; e21pm, exon 21 point mutation; EGFR‐TKI, epithelial growth factor receptor‐tyrosine kinase inhibitor; ORR, objective response rate; PFS, progression‐free survival. ^3^Sequist *et al*. 2013; ^4^Wu *et al*., 2014; ^5^Park *et al*. 2016; ^11^Mayo‐de‐Las‐Casas *et al*. 2017; ^12^Wang *et al*. 2018.

The favorable results observed might be attributable to the dose reduction of afatinib (71%); in fact, AEs of grade ≥ 3 were not as frequently observed as those of the previous LUX‐Lung trials.^3^, ^4^, ^5^ In the present study, the ORR and PFS were higher and longer, respectively, in the 40 mg dose group than in the group with a dose reduction. However, prior trials and real‐world data showed longer PFS in groups with dose reduction than in those without dose reduction.^18^, ^19^, ^20^ Therefore, in a real‐world setting, the survival benefit of afatinib treatment could be achieved not only by using noninvasive assays but also by appropriate dose reduction as determined by frequent and detailed monitoring of toxicities. However, the difference in survival was not proven in randomized controlled trials^18^, ^21^ and in the present study.

The acquisition of the T790M mutation is the main mechanism of acquired resistance to EGFR‐TKIs, including afatinib, regardless of the type of drug, except for third‐generation agents,^22^ where osimertinib is standard second‐line therapy for these patients.^23^ In the GioTag trial, a real‐world study of patients with an acquired T790M mutation after first‐line afatinib therapy, sequential afatinib and osimertinib treatment showed a sustained clinical benefit and could provide a prolonged chemotherapy‐free duration, especially in the Asian population (median treatment time, 46.7 months).^24^ Thus, tests to confirm the T790M mutation have become a mandatory procedure using either tumor rebiopsy or plasma assays after progression following first‐line EGFR‐TKI treatment.

In the current study, rebiopsy or ctDNA analysis (*n* = 9 each) of the T790M resistance mutation was performed in 15 patients after progression, and the T790M mutation was found in 40% (6/15). The detection rate of the T790M mutation was 22% (2/9) in tissue genotyping and 44% (4/9) in ctDNA analysis, and three patients underwent both assays (all of which showed negative results). In our previous study of second‐line osimertinib, 41% (33/80) of the screened patients were positive for the T790M mutation.^25^

In a Canadian validation study of plasma EGFR T790M testing, plasma testing showed more T790M‐positive results (62%) than tumor biopsy alone (49%), with 75% sensitivity using highly sensitive methods such as ddPCR, next‐generation sequencing, and RT‐PCR.^26^ Therefore, ctDNA EGFR analysis could be used in clinical practice as a routine diagnostic tool in a post‐EGFR‐TKI setting and for initial screening at diagnosis.

In conclusion, this study met its primary endpoint to prove a response rate >50% with afatinib in patients with *EGFR* activating mutations detected in ctDNA. In addition, the present study showed a favorable ORR and PFS with afatinib similar to that observed in previous trials assessing it as the first‐line treatment of *EGFR*‐mutated NSCLC based on tumor genotyping. Although we did not design this trial to compare the efficacy of afatinib between Group A (EGFRm in ctDNA only) and B (EGFRm both in ctDNA and tumor DNA), the drug showed similar ORR and PFS in both groups regardless of the tumor EGFRm results.

## Disclosure

There are no conflicts of interest relevant to this article.

## Supporting information


**Appendix S1.** Supporting Information.
