# Supplementary material for: Phase II open‐label multicenter study to assess the antitumor activity of afatinib in lung cancer patients with activating epidermal growth factor receptor mutation from circulating tumor DNA: Liquid‐Lung‐A
Source: Thorac Cancer. 2020 Dec 3;12(4):444–52. doi: 10.1111/1759-7714.13763 (PMC7882376; doi:10.1111/1759-7714.13763)
Supplement: Supplementary file 1 — Appendix S1. Supporting Information. [file TCA-12-444-s001.docx]

**Supporting Information Data**

**Inclusion and Exclusion Criteria**

***Inclusion Criteria***

For inclusion in this study, subjects were required to fulfill the following criteria:

1. Provision of informed consent before any study-specific procedures

2. Age >18 years

3. Presence of locally advanced or metastatic non-small cell lung cancer, not amenable to curative surgery or radiotherapy, with or without pathologic diagnosis

4. No prior exposure to EGFR TKIs (multiple lines of prior cytotoxic chemotherapy are permitted)

5. Activating EGFR mutation (G719X, exon 19 deletion, L858R, L861Q) detected in circulating tumor DNA by PANA Mutyper® test

6. Activating EGFR mutation (exon 19 deletion, L858R, L861Q, G719X) detected from tumor tissue or cytology specimens

7. Eastern Cooperative Oncology Group (ECOG) performance status of 0–2

8. A life expectancy ≥12 weeks

9. Females must be taking adequate contraceptive measures, must not be breast feeding, and must have a negative pregnancy test result before the start of dosing if of child-bearing potential or must have evidence of non-child-bearing potential by fulfilling one of the following criteria at screening: a) post-menopausal, defined as age >50 years and amenorrhoeic for at least 12 months following cessation of all exogenous hormonal treatments, b) women <50 years of age were considered post-menopausal if they had been amenorrhoeic for ≥12 months following cessation of exogenous hormonal treatments and with luteinizing hormone and follicle-stimulating hormone levels in the post-menopausal range for the institution; *c) documentation of irreversible surgical sterilization by hysterectomy, bilateral oophorectomy, or bilateral salpingectomy but not tubal ligation.

10. Male patients should be willing to use barrier contraception.

11. Willingness and ability to comply with the protocol for the duration of the study including undergoing treatment and scheduled visits and examinations, including follow-up

12. At least one lesion not previously irradiated that can be accurately measured at baseline as ≥10 mm in the longest diameter (except lymph nodes, which must have a short axis ≥15 mm), with computed tomography

***Exclusion Criteria***

Participants were not enrolled into the study if they fulfilled any of the following exclusion criteria:

1. Any unresolved toxicities from prior therapy, Common Terminology Criteria for Adverse Events (CTCAE) >grade 1 at study treatment initiation with the exception of alopecia and grade 2 prior platinum-therapy-related neuropathy
2. Any evidence of severe or uncontrolled systemic diseases including uncontrolled hypertension and active bleeding diatheses, which in the investigator’s opinion make it undesirable for the patient to participate in the trial or would jeopardize compliance with the protocol or active infections including hepatitis B, hepatitis C, or human immunodeficiency virus (screening for chronic conditions was not required.)
3. Symptomatic central nervous system metastases that were neurologically unstable
4. Past medical history of interstitial lung disease (ILD), drug-induced ILD, radiation pneumonitis requiring steroid treatment, or any evidence of clinically active ILD
5. Inadequate bone marrow reserves or organ functions, as demonstrated by any of the following laboratory values:

Absolute neutrophil count < 1.5 × 10^9^/L

Platelet count < 100 × 10^9^/L

Hemoglobin levels < 90 g/L

Alanine aminotransferase levels > 2.5 times the upper limit of normal (ULN) if no demonstrable liver metastases or > 5 times the ULN in the presence of liver metastases

Aspartate aminotransferase levels > 2.5 times the ULN if no demonstrable liver metastases or > 5 times the ULN in the presence of liver metastases

Total bilirubin levels > 1.5 times the ULN if no liver metastases or > 3 times the ULN in the presence of documented Gilbert’s syndrome (unconjugated hyperbilirubinemia) or liver metastases

Creatinine levels > 1.5 times the ULN, concurrent with creatinine clearance < 50 mL/min (measured or calculated using the Cockcroft and Gault equation); confirmation of creatinine clearance was only required when creatinine levels were > 1.5 times the ULN

1. Any of the following cardiac criteria:
2. Mean resting corrected QT interval (QTc using Fridericia’s formula) > 470 ms
3. Any clinically important abnormalities in rhythm, conduction, or morphology identified in a resting ECG (e.g., complete left bundle branch block, third-degree heart block, second-degree heart block)
4. Any factors that increase the risk of QTc prolongation or arrhythmic events such as heart failure, hypokalemia, congenital long QT syndrome, family history of long QT syndrome, unexplained sudden death at <40 years of age in first-degree relatives, or any concomitant medication known to prolong the QT interval
5. Refractory nausea and vomiting, chronic gastrointestinal diseases, inability to swallow the formulated product, or previous significant bowel resection that would preclude adequate absorption of afatinib
6. History of hypersensitivity to afatinib (or drugs with a similar chemical structure or class to afatinib) or any excipients of these agents
7. Male and female patients of reproductive potential who are not using an effective method of birth control and female patients who are pregnant, breastfeeding, or have a positive (urine or serum) pregnancy test result before study entry
8. Judgment of the investigator that the patient should not participate in the study if they are unlikely to comply with study procedures, restrictions, and requirements
9. Previous allogeneic bone marrow transplantation
10. Non-leukocyte-depleted whole blood transfusion within 120 days of the date of the genetic sample collection
